# Supplementary material for: Prevalence of neurocysticercosis and its characteristics among people with epileptic seizures and progressively worsening severe headaches in 60 villages in three provinces of Burkina Faso
Source: PLoS Negl Trop Dis. 2026 Feb 26;20(2):e0014061. doi: 10.1371/journal.pntd.0014061 (PMC12962496; doi:10.1371/journal.pntd.0014061)
Supplement: Attachment — Submitted filename: Revision Note - Final.docx [file pntd.0014061.s002.docx]

**Revision Note: PNTD-D-25-00377**

Dear Editor and Reviewers of PLoS Neglected Tropical Diseases,

We wish to thank you for your time and your thorough review of our manuscript. Please find our responses to the reviewers’ comments and changes suggested below. All changes made within the manuscript were marked using “track changes”.

Thank you again,

Best regards,

The authors of the manuscript.

**Reviewers' comments:**

**Reviewer #1:**

1) good study, nicely analyzed

Thank you for your kind words.

**Reviewer #2:**

1) Prevalence of neurocysticercosis and its characteristics among people with epileptic seizures and progressively worsening severe headaches (PWSH) in 60 villages in three provinces of Burkina Faso

The manuscript reports part of the findings of a study on NCC in 60 villages in three provinces of Burkina Faso. It estimated NCC prevalence in progressively worsening severe headaches (PWSH) in the rural regions of Burkina Faso. Human neurocysticercosis (NCC) in sub-Saharan Africa (SSA) is recognized as a significant but under-diagnosed and under-reported public health issue. Most of our knowledge comes from specific research projects or hospital settings, which may not represent the general population. The study addresses gaps in NCC epidemiology in sub-Saharan Africa, particularly Burkina Faso, where data from the general population are limited. However, several issues should be addressed by the authors before considering the manuscript for publication.

Thank you for your thorough review of our manuscript. We recognize that our study was not perfect, but our revisions based on your comments and our associated responses, will hopefully help clarify several issues, provide explanations and emphasize limitations that must be taken into account when interpreting these results. Please find below our answers to your concerns

2) This work is the latest of the several publications derived from a project conducted in 2011-2012. The authors are expected to elaborate more about the aspects of NCC in Burkina Faso that are new in this report, and what data were NOT covered in the past six publications.

Thank you for this question. In the earlier manuscripts reporting on data of the project, no detailed characterization was given of the neurocysticercosis (NCC) cases identified in the project, nor was the prevalence of neurocysticercosis in people with progressively worsening severe headaches or epilepsy estimated. Very few studies have so far reported the proportion of NCC among people with headaches, a symptom increasingly recognized as associated with NCC. Moreover, the proportion of NCC among people with epilepsy is important to gage what impact interventions targeting cysticercosis could have on this stigmatizing outcome in Burkina Faso. Hence, our manuscript reports on several aspects not previously covered in the other publications.

3) - According to the manuscript, the aim of the study was to describe the types of NCC lesions seen on cerebral computed tomography (cCT), however, only symptomatic individuals (epilepsy/PWSH) were studied, and the study excluded asymptomatic NCC cases. in addition final diagnosis was based on physician notes review, not direct patient examination, and this risks misclassification. This presents a selection and/or classification bias. Please justify exclusion of asymptomatic individuals and address potential selection bias. Also, the distinction and potential misclassification of “uncertain” NCC cases is underdeveloped.

Clarification of Study Aim and Study Population: The study aimed to characterize the cerebral CT findings and estimate the proportion of NCC among individuals with epilepsy and/or progressively worsening severe headaches (PWSH) in 60 villages in Burkina Faso. As correctly pointed out by the reviewer, this aim does not include asymptomatic individuals. To ensure clarity about our aims, we have now revised the wording in the Abstract, Introduction, Methods to explicitly state that the neuroimaging-based lesion characterization pertains to individuals with confirmed symptomatic presentations (epilepsy and/or PWSH) only as follows:

Abstract: *Our aim was therefore to describe the types of NCC lesions seen on cerebral computed tomography (cCT), among participants with PWSH and epilepsy from 60 participating villages of Burkina Faso, and to determine the prevalence of NCC in these groups.*

Introduction: *The present study aimed to describe the types of NCC lesions seen at neuroimaging among individuals with epilepsy and PWSH in these 60 villages in Burkina Faso, and estimate the proportion of NCC in these groups.*

Methods: *For the present study, the study sample entails those participants of the baseline cross-sectional component of the trial with confirmed neurological signs/symptoms of interest (epilepsy and PWSH) who underwent cCT, allowing us to describe the types of NCC lesions seen at neuroimaging among individuals with in these 60 villages in Burkina Faso, and estimate the proportion of NCC in these groups. Participant selection for the present study was based on a two-step process: a screening questionnaire followed by diagnostic confirmation by physicians/neurologists.*

Justification for Exclusion of Asymptomatic Individuals: Inclusion was limited to symptomatic individuals for ethical reasons. Exposing healthy individuals to the radiations of cCT is not ethical, and therefore was not done. Moreover, our focus was to describe lesion patterns in individuals suffering from the burden of neurological signs/symptoms that may be caused by NCC, and not to estimate NCC prevalence in the general population. These justifications are now clarified in the Discussion, and we have elaborated more on the generalizability in the Limitations as well:

Discussion: *This study aimed to describe the type of NCC lesions seen on neuroimaging in people with epilepsy and PWSH in 60 villages in Burkina Faso, and to estimate the prevalence of NCC in these groups. Few community-based studies of this scale have investigated the contribution of NCC to epilepsy, and even less that to PWSH in endemic areas, making this estimation clinically and programmatically relevant. This is especially important since epilepsy and PWSH are the most common neurological manifestations of symptomatic NCC (1) and result in important monetary and societal burden for the patients and their family (31-33). Unravelling the frequency of NCC in these subgroups will therefore assist in informing differential diagnostic decision-making, in prioritizing resource allocation (e.g. neuroimaging and treatment), and in designing targeted interventions in endemic settings. Although estimating the prevalence of NCC in the general population would be of interest, it is unethical to conduct cCT among individuals who do not present with neurological signs/symptoms. In addition, such information would have limited immediate clinical utility and not directly benefit participants.*

Limitations: *By design, we focused on the occurrence of NCC in participants with the neurological signs/symptoms of interest. Hence these results are not meant to be generalizable to the general population.*

Addressing Potential Selection and Classification Bias: The validity (verification bias and misclassification error) of the two-step approach (screening questionnaire followed by diagnostic confirmation by the physicians/neurologist) has been described in great detail in the paper of Sahlu et al. 2019 (doi 10.1371/journal.pntd.0007109). However, it is important to underline that the two physicians who conducted the physical and neurological examinations in the field were contacting the neurologist by phone when they were uncertain about their diagnostic. In addition, the non-perfect nature of the clinical diagnostic of seizures and epilepsy is well known, especially a community-based setting where there is no access to video EEG. We have now reflected further on this in the Limitations section:

*Furthermore, the diagnosis of potential NCC-related signs/symptoms, such as epilepsy and PWSH is not straightforward, especially in a community-based setting. First, and as shown by our team (21), the use of a screening questionnaire for the neurological signs/symptoms of interest has its limitations and can lead to missing cases suffering from these signs/symptoms. Indeed, the screening questionnaire was estimated to have a sensitivity and specificity of 66.1% and 88.9% for epilepsy and 59.6% and 88.6% for PWSH, respectively (21). Second, we assumed that the evaluation by the study physicians/neurologist was a reference standard for establishing the presence of the signs/symptoms of interest, which is not the case, especially in a community-based setting without access to a video electroencephalogram (EEG) (42) or standard definitions for severe headaches resulting from NCC. Third, when the study was conducted and up to now, a standard definition and associated standardized questionnaire for NCC-associated headaches have been lacking (13). Hence, the assessment method for PWSH had its limitations as the definition used in the study was more geared towards chronic headache as defined in the context of primary headache. Fourth, the approach for the final neurological diagnosis was based on the evaluation of the neurologist reviewing the original notes of the physicians who had examined the patients. This means that the neurologist also did not examine the patients himself, which has its drawbacks and can compound classification error. However, in this large community-based randomized controlled trial, the estimation of the prevalence of NCC among people with epilepsy and PWSH was a secondary objective, and hence, sending all people diagnosed by the study physicians for routine video EEG or additional examination by the neurologist, was not feasible with the available funds and with the limited resources in public hospitals of Burkina Faso in 2011. Moreover, we observed a perfect agreement in the diagnostic evaluation from the physicians for 35 individuals who were examined during the two rounds of visits for the medical examinations (21).*

Clarification on “Uncertain” NCC Cases: We appreciate the reviewer’s point regarding the categorization of “uncertain” NCC cases and have expanded our explanation in the Methods. Specifically: “*Individuals with possible NCC lesions on neuroimaging which did not meet radiological criteria for definitive or probable NCC according to the diagnostic criteria (4), were categorized as uncertain NCC. To reflect the diagnostic uncertainty, and because we consider them neither confirmable as NCC case nor were they confidently ruled out, these cases were described separately from the definitive and probable cases.*”

4) - please provide more details of CT imaging protocols (slice thickness, contrast use) and radiologist consensus process.

We have now added more details on the slice thickness and the contrast use in the Methods section:

*All confirmed epilepsy and PWSH cases were invited to undergo cCT, performed using a General Electric 8 Barrett LightSpeed scanner (General Electric) at the Centre Médical Schiphra in Ouagadougou. We performed a biphasic helical acquisition, both without and with injection of iodinated contrast medium, using native slices of 5 mm thickness, multiplanar reconstructions, and image readings in parenchymal and bone windows. Telebrix 35 (50 ml; Guerbet, Aulnay-sous-Bois, France), the only contrast medium available in Burkina Faso at the time, was used as iodinated contrast medium. It was administered intravenously, with image acquisition 5–10 minutes later to allow for adequate cerebral enhancement. Prior to the injection, the renal function was verified by measuring serum creatinine levels and calculating the glomerular filtration rate (GFR) using the Cockcroft–Gault formula (27). All patients had a GFR greater than 30 mL/min/1.73 m², allowing the injection of contrast medium without risk of contrast-induced nephropathy. No participant experienced an allergic reaction to the iodinated contrast medium.*

Regarding the radiologist consensus process, the cCT scans were independently reviewed by three neuroradiologists (RC, MPB, VR) until consensus was reached. When a consensus could not be reached, the cCT scans were further evaluated by a neurologist (ASW) with extensive experience in NCC.

5) - Delayed CT participation due to epilepsy stigma may bias prevalence estimates. Report attrition rates, e.g., how many refused CT scans due to stigma?

We wish to thank the reviewer for raising this relevant point. As noted in the manuscript, the study team applied various approaches to try and reduce the impact of stigmatization on the participants, including having the physicians run multiple visits, spread over time, to allow him to examine all people who had screened positive for the neurological signs/symptoms of interest. Cases confirmed with the neurological signs/symptoms of interest were invited to travel to another location to take a bus to the capital for cCT. Moreover, the delay was also due to people not being present at the time of the physician’s visit to the village, due in part to high work migration patterns and possibly stigma and fear of being examined, requiring a second “catch up” visit several months later. This was not a clinical-based study, rather, it was a community-based study in rural areas and among a very marginalized population, these delays are to be expected. Getting to some villages which were only accessible by small dirt trails was challenging and also explains some of these delays.

Unfortunately, precise attrition figures due specifically to stigma-related refusal were not systematically recorded, and this may not have been possible. This study did not focus on stigma caused by neurological disease, something which is very challenging to measure. In addition, those suffering from stigma will probably not admit to it because of that stigma. If someone is stigmatized, they are unlikely to tell a research team that they are and that it is the reason why they will not present to an appointment for cCT.

However, among the 336 individuals who were confirmed to suffer from the neurological signs/symptoms of interest who were scheduled for neuroimaging, 249 (74%) ultimately underwent cCT. We have now clarified this in the Results.

6) Findings from 3 provinces out of 45 provinces in the country may not reflect Burkina Faso’s broader epidemiology.

The primary objective of this study was to estimate the effectiveness of an educational programme to control human and porcine cysticercosis. As such, the sampling strategy was not designed to be representative of the whole country, but rather of the villages that met our selection criteria. Our study areas were selected based on pig-raising practices and rural population density to align with the objectives of the broader cluster randomized trial which had to be conducted in an area endemic for cysticercosis. While this sampling limits national generalizability, the provinces included are among the more endemic and relevant regions for *Taenia solium* transmission. We have now explicitly acknowledged this limitation in the Discussion, emphasizing that our findings reflect regional patterns in high-risk, low sanitation, pig-raising areas rather than the entire country:

*Due to our sampling strategy focusing on including participants from 60 villages in three provinces believed endemic for cysticercosis, our findings are not representative of the entire country, but rather reflect the patterns in high-risk, pig-raising villages with poor sanitation that met the inclusion criteria of our sampling frame.*

7) - Only descriptive statistics are used, despite the availability of rich demographic and clinical data. Please consider conducting logistic regression to more in-depth information.

We appreciate the reviewer’s suggestion and agree that additional analyses such as logistic regression could yield further insights. However, the primary aim of this study was to provide a descriptive overview of the NCC lesions among individuals with epilepsy and PWSH and to estimate the NCC prevalence in these groups. As such, the scope was intentionally limited to descriptive statistics to remain aligned with this objective.

8) - Median 31–99 days between screening and CT may have altered lesion visibility (e.g., resolved cysts). The limitations should be further emphasized in the context of community-based neuroimaging studies.

We agree that the time delay between screening for the neurological signs/symptoms of interest and cCT neuroimaging could have influenced the presence of lesions and the stage of the cysts. We have now further emphasized this beyond what was already mentioned in the Discussion:

*For some cases, there was also quite a considerable time interval between the screening questionnaire and cCT due to several reasons, including important population movements (17,43) and to the stigma linked to epilepsy as mentioned above (44). Hence, for some participants, the neuroimaging results might have changed as compared to those that would have been observed around the time of administration of the screening questionnaire and of the clinical and neurological evaluation by the physicians, including the possible disappearance of some lesions. This delay may have contributed to the underestimation of viable or degenerating cysts and an overrepresentation of calcified lesions, thereby affecting the clinical classification of NCC. Some cysts could have also completely resolved, resulting in an overall underestimation of the prevalence of NCC in the study population. Such temporal gaps are a common challenge in community-based neuroimaging studies conducted in resource-limited and stigma-prone environments, and should be taken into account when interpreting prevalence and lesion type estimates.*

9) - Please justify the rationale for including PWSH. This is only briefly mentioned in the Introduction.

PWSH was included as a symptom of interest based on evidence suggesting that chronic, severe headaches can be associated with symptomatic NCC. In clinical series it was the second commonest manifestation. While headache is a non-specific symptom, previous studies have reported a higher prevalence of NCC among individuals with persistent or crescendo/decrescendo headaches in endemic settings. In fact, in a recent meta-analysis conducted by our research team, and which is now in preparation for submission to PLoS NTDs, we found that the pooled proportion of headaches as a presenting symptom among people presenting with NCC (diagnosed with neuroimaging) has increased in the past 15 years and was 45% (95% Bayesian Credible Interval: 35%-55%) for studies conducted between 2008 and 2023. This is in contrast to an estimated 37% (95% BCI: 29%-45%) between 1990 and 2008. In addition, our team only found three studies meeting our rigorous inclusion criteria which estimated the proportion of NCC among people with headache (also a manuscript soon to be submitted). We could not generate a pooled estimate due to important variation in the methods used and the study populations. One neurology/headache clinic-based study reported the proportion of NCC among people with primary headaches at 4.8% (Bayesian Credible Interval (BCI): 3.6%-6.2%) (Del Brutto, 2012 (doi: 10.1177/0333102411433043.)). The only community-based studies which met our inclusion criteria, conducted in Ecuador in 1992, reported a proportion of 28% (95% Bayesian Credible Interval (BCI): 18.4%-39%) of NCC among people with severe headache (Cruz et al., 1995 (doi: 10.1111/j.1526-4610.1995.hed3502093.x)). This clearly demonstrates that there is a dearth of evidence on the frequency of NCC among people with different types of headache

We have now adapted the Introduction as follows:

*Headaches have been reported as the second commonest manifestation of symptomatic NCC in patients seen in neurology clinics, as found in another systematic review (1). However, we are aware of only one study with low risk of bias reporting the proportion of NCC lesions among people with severe headaches in a community-based setting. That study was conducted in a rural community of Ecuador in 1992 where the proportion of NCC detected by CT was 28% (Bayesian Credible Interval: 18.4%-39%) among people with migraine or tension headaches (14).*-

10) The link between lesion type (e.g., calcified vs. active) and clinical symptoms is discussed but NOT statistically explored. Please elaborate more on calcified lesion dominance vs. active cysts in relation to seizure/headache mechanisms.

In line with our response above, as the primary aim of this study was to provide a descriptive overview of the NCC lesions among individuals with epilepsy and PWSH and to estimate the NCC prevalence in these groups, we feel these additional analyses would be beyond the scope our paper.

11) - Please replace the Discussion with "conclusion".

As per PLoS NTD guidelines on manuscript organization, we have opted to keep the section heading as Discussion instead of Conclusion.

12) - The citation for Jensen & Stovner (line 138) is not correct (26 > 25).

This is correct, “Jensen and Stovner (2008)” has now been adapted to “Jensen and Stovner (23)”.

**Reviewer #3:**

1) The aim of this study was to assess the relationship between symptoms possibly related to NCC (epilepsy and headache) and the diagnosis of NCC. While this may be interesting, there are serious problems with the methodology, the analysis of the results is not complete, and the article lacks relevant conclusions.

Thank you for your thorough review of our manuscript. We’ve tried to address the raised comments as much as possible. Please find our responses below.

2) In this door-to-door study, a questionnaire relating to neurological symptoms (epilepsy, PWSH) was applied to residents of rural communities. 109 people were diagnosed with epilepsy, 116 with PWSH and 15 with both symptoms. A CT scan with/without contrast was carried out in all these people (240), and in 30 of them a definite (17) or probable (13) diagnosis of NCC was made. The main problem is the definition of PWSH (lines 133-136). This definition could possibly be compatible with headache in the context of ICH, but not in the context of other NCC presentations. I really don't know on what basis the authors consider this type of headache to be a primary symptom of NCC. It isn't. I'm really very surprised that so many individuals present with this type of symptomatology. Since the individuals included have not been evaluated by a neurologist, serious doubts about the diagnosis can be raised. Headaches are the most common neurological symptoms, and many causes exist, particularly tension headaches. The authors do not specify whether the individuals who suffered from this symptom attended the health center, but if they really did suffer from this type of headache, it is very likely that they received medical attention. No information on this point is available.

Thank you for your comment. This study was conducted in marginalized, often isolated, resource-poor populations in rural Burkina Faso in 2011 where and when access to health care was very limited. In addition, when conducting the neurological examination, many participants really had difficulty rating their pain, and did not understand the typical tool of faces to grade it. Assuming that people would seek medical attention in this type of environment is incorrect. In addition, at the time of this study, headaches had not been much described in NCC, and our definition, adapted from the definition by Jensen and Stovner was what seemed to be best matching the types of headache associated with NCC described in the literature. This definition was proposed by our colleague, and now deceased, neuro-epidemiologist Dr. Linda D Cowan. We have provided our definition for PWSCH and report the results which we found in all transparency.

We would like to also clarify that this was not a “door-to-door survey” as the participants were selected according to the concessions they lived in and one person per concession was selected at random. As mentioned in previous answers, this was the baseline component of a clustered randomized trial, and not a population survey to estimate the prevalence of epilepsy or PWSH.

3) The main results of this study are as follows:
   - The prevalence of NCC (def or probable) in symptomatic individuals is 12.5% (30/240), 7.1% if only definitive diagnosis of NCC is considered.
   - The prevalence of NCC (def or probable) in subjects suffering only from headaches is 5.2% (6/116), with only one individual having a definitive diagnosis of NCC (prevalence: 0.8%).
   - The prevalence of NCC (def or probable) in epileptic subjects is 18.3% (20/109), 12% (12/109) if only definitive diagnosis of NCC is considered.
   - The prevalence of NCC (def or probable) in subjects with headache + epilepsy is 26.7% (4/15), all with a definitive diagnosis of NCC.

Unfortunately, the authors do not clearly express these results, and the article lacks clear conclusions.

We thank the reviewer for reflecting on our main findings. We appreciate this opportunity to clarify the way our prevalence estimates were derived, as there may have been some differences in how the denominators were interpreted.

In our study, prevalence was calculated among participants with confirmed neurological signs/symptoms of interest who underwent cCT. Because our goal was to estimate the prevalence of NCC within each clinical manifestation, individuals presenting with both epilepsy and PWSH were included in both groups. Consequently, the denominators were 126 for epilepsy and 131 for PWSH, giving a total of 249 participants with valid cCT results. The figures mentioned by the reviewer (e.g., 240 participants) appear to result from summing the three symptom categories—epilepsy, PWSH, and both—as mutually exclusive, thereby excluding a small number of participants and slightly changing the proportions. Using overlapping categories, as specified in our analysis plan, results in the following prevalence estimates:

Among people with epilepsy (n = 126): 21 definitive or probable NCC cases → 16.9% (95% CI 10.8–24.7).

Among people with PWSH (n = 131): 10 definitive or probable NCC cases → 7.6% (95% CI 3.7–13.6).

These denominators and the rationale for counting individuals with both symptoms in each subgroup were described in the Results section (“Prevalence of the neurological signs/symptoms of interest among NCC cases and link with radiological findings”), however to enhance clarity for readers, we have now indicated explicitly in the Results section that overlapping symptom groups were analyzed in both categories:

*For clarity, individuals who presented with both epilepsy and PWSH were included in both the epilepsy and PWSH categories, as the objective was to estimate the prevalence of NCC within each clinical manifestation rather than in mutually exclusive groups. Consequently, the denominators for these prevalence estimates were 126 for epilepsy and 131 for PWSH, yielding a total of 249 participants with valid cCT results.*

4) Lines 50-52. Regional variation? This point and its possible causes are not thoroughly discussed in the article.

What is meant by this statement in the Abstract, is that there is a considerable variation between countries in terms of the NCC prevalence estimates in people with epilepsy. This has been mentioned in the discussion by highlighting the wide range in NCC prevalence estimates detected in groups with the neurological signs/symptoms of interest.

5) Lines 77-78: False. In the systematic review mentioned by the authors (the same corresponding author of this work), headaches (and not PWSH) were indeed the second most frequent symptom. But we are talking about two completely different entities. Moreover, as the authors mention in ref. 3, “These estimates are only applicable to patients who are ill enough to seek care in neurology clinics and likely overestimate the frequency of manifestations among all NCC cases”. No information on attention in a health center of the included individuals is available in this article.

We agree that the systematic review referred to in reference (1) reported headaches broadly—not progressively worsening severe headaches (PWSH)—as the second most frequently observed symptom among symptomatic NCC cases. We acknowledge that these are distinct clinical entities and that our wording inaccurately implies an equivalence. We have now revised the sentence to more accurately reflect the evidence, stating: “*Headaches have been reported as the second commonest manifestation of symptomatic NCC in patients seen in neurology clinics, as found in another systematic review (1).*” In addition, as mentioned above, our study was conducted in very marginalized, isolated and poor populations who did not have the resources to seek medical care.

6) Lines 78-80. Headaches are one of the most common neurological symptoms and are not really relevant to the diagnosis of NCC, as evidenced by your results.

It is true that headaches are a common neurological complaint with many potential causes, and are therefore non-specific in the context of NCC diagnosis. Our intent in this sentence was not to suggest that headaches alone are diagnostic of NCC, but rather to highlight that, in some cases, headache may be part of the clinical picture of symptomatic NCC. Nevertheless, to avoid confusion, we have now dropped this statement.

7) Lines 157-158. Contrast injection can cause kidney damage. The authors did not check renal function beforehand. This is really a mistake, especially as some of the people included were over 50, and their medical history was unknown.

Thank you for pointing out the omission of providing details regarding the potential kidney damage of contrast injection. Kidney function was checked before contrast injection. We have now adapted the text in the Methods section as follows (whilst also addressing the request of Reviewer 2 for more details on the cCT protocol):

*All confirmed epilepsy and PWSH cases were invited to undergo cCT, performed using a General Electric 8 Barrett LightSpeed scanner (General Electric) at the Centre Médical Schiphra Center in Ouagadougou. We performed a biphasic helical acquisition, both without and with injection of iodinated contrast medium, using native slices of 5 mm thickness, multiplanar reconstructions, and image readings in parenchymal and bone windows. Telebrix 35 (50 ml; Guerbet, Aulnay-sous-Bois, France), the only contrast medium available in Burkina Faso at the time, was used as iodinated contrast medium. It was administered intravenously, with image acquisition 5–10 minutes later to allow for adequate cerebral enhancement. Prior to the injection, the renal function was verified by measuring serum creatinine levels and calculating the glomerular filtration rate (GFR) using the Cockcroft–Gault formula (27). All patients had a GFR greater than 30 mL/min/1.73 m², allowing the injection of contrast medium without risk of contrast-induced nephropathy. No participant experienced an allergic reaction to the iodinated contrast medium.*

8) Line 171. In reference 6, the definition of “uncertain NCC cases” does not exist. These cases are not NCC and should be included in the results section as “non NCC cases”.

See our response higher up regarding our justification to present the data on the “uncertain NCC” cases. We feel they are neither confirmable as NCC nor can they be confidently ruled out.

9) Line 184. PDA? Explain

This abbreviation (PDA, Personal Digital Assistant) was earlier explained in the last sentence of the section ”*Measurement and definitions of epilepsy and progressively worsening severe headaches*”. We have now expanded the wording by indicating that it is a “*handheld device for information storage*”. PDAs were popular for data entry in the field before the boom of smartphones and tablets.

10) Line 200: “as at the time of the study, the final diagnosis was not yet known”. I don't understand. Was the scanner not part of the study? This is not at all clear.

We thank the reviewer for this observation and agree that the sentence was unclear. The cCT was indeed part of the study procedures, performed to help establish the final diagnosis. At the time of providing symptomatic treatment, however, the results of the cCT scans were not yet available, which is what we meant by “as at the time of the study, the final diagnosis was not yet known.” We have revised the text in the Methods section to clarify this point.

*As an incentive to participate in the study, all participants were offered a bar of soap. Additionally, all patients received standard care according to national guidelines. Because the neuroimaging results were not available at the time of the examination by the physicians, symptomatic treatment was provided based on the presenting neurological signs and symptoms. Participants with PWSH were provided with paracetamol for a few weeks, whereas participants with epileptic seizures received phenobarbital for at least 6 months. During this period, the field team was around and in close contact with the involved nurses in the study villages, who monitored any side effects of the medication and contacted the medical team for assistance or advice as needed.*

11) Line 207. 249 out of how many people evaluated?

Thank you for your comment. We agree that this was unclear. We have added the following information in the first part of the Results section:

*A total of 4,970 people were screened for epileptic seizures and PWSH, among whom 880 individuals were examined by one of the two physicians in the field. Of those, 336 were confirmed to suffer from the neurological signs/symptoms of interest, and 249 individuals (74%) underwent cCT examination (…).*

12) Line 208. The neurologist never saw the patient. Although the authors report this problem as a limitation, it is a reason to invalidate the interest of this study.

This approach is indeed an important limitation of our study, but in line with the reality of many resource-poor settings. However, the physicians had been trained by the neurologist for the diagnostic of epilepsy, epileptic seizures and PWSH. In addition, the physicians called the neurologist whenever he had doubts about the diagnosis. However, we do recognize that this is a limitation, and have now expanded the discussion regarding this limitation as follows:

*This entailed that the neurologist also did not examine the patient himself, which has its drawbacks and can compound classification error.*

However, our estimates of the prevalence of NCC among PWE is in line with a meta-analysis which we are preparing for submission to PLoS NTDs and which found a pooled estimate of 17% (95BCI: 11% to 24%) for studies conducted between 2008 and 2023 such as ours, which completely agrees with our results. This is a strong argument in favour of validating the interest of this study.

13) Lines 223-226. The results of Ag and Ab detection were part of the diagnostic criteria. These sentences are therefore of little interest.

The Ag and Ab test results were not part of the criteria to differentiate between definitive/probable case on the one hand, and the uncertain cases on the other hand. As mentioned above, what we consider as “uncertain” NCC cases, are those with lesions for which the radiologists were unsure whether they were suggestive for NCC or not. This group was not evaluated against the revised Del Brutto criteria. Only those with the definitive and probable NCC group was the classification as definitive or probable NCC cases based on the Del Brutto criteria, including the Ab and Ag test results, applied. Please also kindly refer to our response higher up about the “uncertain” NCC cases.

14) Lines 230-231. Did the CT scan detect any other brain lesions other than NCC? Please give details. Has surrounding edema or contrast enhancement been detected around any calcified cases?

Due to inconsistencies in the data collection for other brain lesions (considering that it was not our primary objective to report on these lesions) as well as missing data, we prefer not to report on other brain lesions. As for perilesional edema around calcifications, the CT equipment available was not performant enough to reliably measure it.

15) Table 1. Only one patient with PWSH has been definitively diagnosed with NCC. This should be discussed.

Thank you for noting this. This observation has now been added to the Results:

*Of note, only one patient with PWSH was been definitely diagnosed with NCC.*

16) How do the authors explain that none of the three individuals with hydrocephalus suffered from headaches? This is really strange and raises doubts about the proper assessment of “PWSH”. What therapeutic measures were taken for these patients?

We thank the reviewer for this insightful observation. We agree that the absence of headaches among individuals with hydrocephalus may seem unexpected. However, hydrocephalus is not invariably associated with headaches. The clinical presentation depends on the chronicity of the condition, the rate of cerebrospinal fluid accumulation, and the degree of intracranial pressure compensation. In chronic or compensated forms, intracranial pressure may remain stable, and patients can remain asymptomatic or present with only subtle or non-specific symptoms (Tullberg et al., 2024 (DOI: 10.1016/j.wneu.2023.12.094)).

All three individuals with hydrocephalus identified in our study were clinically stable at the time of evaluation and did not present signs of raised intracranial pressure or neurological deterioration. Therefore, no neurosurgical intervention (e.g., shunt placement) was indicated. They received symptomatic management in accordance with national clinical guidelines and were followed up by the study team and local health personnel. In addition, these patients were advised to seek consultation with the neurosurgery team located in the capital city, in order to obtain specialized evaluation and guidance for appropriate long-term management.

17) Table 2 - Not relevant, as these people do not meet the NCC diagnostic criteria.

See our response higher up regarding the justification to present data on what we classify as “uncertain” NCC cases.
