# Supplementary material for: Prevalence of neurocysticercosis and its characteristics among people with epileptic seizures and progressively worsening severe headaches in 60 villages in three provinces of Burkina Faso
Source: PLoS Negl Trop Dis. 2026 Feb 26;20(2):e0014061. doi: 10.1371/journal.pntd.0014061 (PMC12962496; doi:10.1371/journal.pntd.0014061)
Supplement: Attachment — Submitted filename: Revision_Note_-_Final_auresp_2.docx [file pntd.0014061.s003.docx]

**Revision Note: PNTD-D-25-00377R2**

Dear Editor and Reviewers of PLoS Neglected Tropical Diseases,

We wish to thank you again for your time and thorough review of our revised manuscript. Please find our responses to the new reviewers’ comments and changes suggested below. All changes made within the manuscript were marked using “track changes”. We hope you will now find the manuscript acceptable for publication.

Thank you again,

Best regards,

The authors of the manuscript.

**Reviewers' comments:**

**Reviewer #2**

1) Almost all the comments are appropriately addressed in the manuscript, thanks, no further comments.

Many thanks for your review of our paper.

**Reviewer #3**

1) Although the authors have made efforts to address my concerns, several important issues remain unresolved.

Thank you for your thorough re-evaluation of our manuscript. We appreciate your acknowledgement of our efforts to address your previous comments and understand that several important issues remain unresolved. In this newly revised version, we have sought to clarify these points, provide additional explanations where needed, and more clearly acknowledge the limitations that should be considered when interpreting our findings. Please find below our detailed responses to each of your concerns.

2) I still do not fully understand the rationale behind the “uncertain” diagnostic category. The authors state: “Individuals with possible NCC lesions on neuroimaging which did not meet radiological criteria for definitive or probable NCC according to the diagnostic criteria (4) were categorized as uncertain NCC.” Diagnostic criteria for NCC have been carefully developed by multiple experts, and introducing a new category that is not part of these internationally accepted criteria is not appropriate. What does this new category contribute to the results, and what is its relevance? The statement in the abstract (lines 45–46) is inaccurate: this category is not in accordance with the “internationally recognized diagnostic criteria for NCC.” This category must be deleted.

As written in the text, we consider these cases neither confirmable as NCC case nor were they confidently ruled out, therefore these cases were described separately from the definitive and probable cases. Nevertheless, we acknowledge that the wording could have been better. In fact, these cases had lesions on neuroimaging for which the radiologists were unsure whether they were indeed cystic lesions caused by NCC, therefore they could not confidently say whether the major diagnostic criterion was met or not.

In the Abstract, we have revised the following statement:

“*These individuals were finally clinically categorized as definitive, probable or uncertain cases of NCC according to the internationally recognized diagnostic criteria for NCC*.“

Into

“*These individuals were finally clinically categorized as definitive or probable cases of NCC, according to the internationally recognized diagnostic criteria for NCC or as uncertain cases of NCC when radiologists were unsure about the lesions*.”

In the Methods, we have revised the following explanation:

*“Observed lesions were radiologically categorized as lesions highly suggestive of NCC, characterized as either active (viable cysts: vesicular stage with or without scolex; degenerating cysts: colloidal or granular nodular stage) or inactive (calcified stage); or possible NCC lesions, in case the radiologists were unsure (28,29).”*

into

“*Observed lesions were radiologically categorized as lesions highly suggestive of NCC, characterized as either active (viable cysts: vesicular stage with or without scolex; degenerating cysts: colloidal or granular nodular stage) or inactive (calcified stage) (28,29); or uncertain NCC lesions, in case the radiologists were unsure they met the descriptions included in the neuroimaging criteria for NCC diagnosis (4).”*

And

“*Individuals with possible NCC lesions on neuroimaging which did not meet radiological criteria for definitive or probable NCC according to the diagnostic criteria (4), were categorized as uncertain NCC*.”

As

“*Individuals with uncertain NCC lesions, i.e. lesions for which the radiologists were unsure whether they met the descriptions of the neuroimaging criteria for NCC diagnosis (4), were categorized as uncertain NCC cases*.”

3) The authors claim that performing contrast CT scans on asymptomatic individuals is not ethical, but this appears to have been done. In Table 1, among the individuals classified as definitive + probable NCC, two subjects reported as symptomatic at first evaluation were later considered asymptomatic by a neurologist. The same applies to one participant categorized as “uncertain” NCC, and it is unclear how many individuals with normal CT scans fall into this situation.

The claim you are referring is related our response and related edits to a previous request for clarification regarding the exclusion of asymptomatic individuals for cCT. As mentioned then, our aim was to describe the types of NCC lesions seen on cCT, among participants with PWSH and epilepsy from 60 participating villages of Burkina Faso, and to determine the prevalence of NCC in these groups. Although estimating the prevalence of NCC in the general population would be of interest, it is indeed unethical to conduct cCT among individuals who do not present with neurological signs/symptoms. In addition, such information would have limited immediate clinical utility and not directly benefit participants.

As mentioned in our Methods, all participants initially diagnosed as having epilepsy or SCWH based on the physicians’ evaluation were invited to undergo cCT. Subsequently, for quality assurance and to ensure consistent application of strict academic definitions, the neurologist reviewed the physician’s clinical notes and strict case definitions. This secondary review resulted in a small number of participants (two initially classified as definite/probable NCC and one initially classified as “uncertain” NCC) being reclassified as not meeting the strict definition of epilepsy and/or PWSH. Importantly, these participants were not randomly selected from the community nor invited for cCT in the absence of neurological complaints; rather, they underwent cCT based on the initial clinical assessment indicating neurological manifestations at the time of enrolment.

We have now clarified this by editing (amongst others), in the Methods:

“*All confirmed epilepsy and PWSH cases were invited to undergo cCT*”

into

“*All participants confirmed to suffer from epilepsy and PWSH based on the physicians’ evaluation, were invited to undergo cCT*”

4) Moreover, the added value of contrast CT is never discussed. The authors do not explain how contrast-enhanced CT contributed beyond non-contrast CT, which is particularly relevant given the discussion regarding the relationship between epilepsy, calcifications, and inflammation.

Thank you for this insightful comment. Indeed, our analysis did not include a blinded comparison between non-contrast and contrast-enhanced CT. However, contrast enhancement plays an important role in the established diagnostic framework for neurocysticercosis: the detection and follow-up assessment of enhancing lesions are part of the major diagnostic criteria, and abnormal enhancement of the basal leptomeninges forms part of the minor criteria (reference #4). In addition, contrast administration is routinely indicated when evaluating any indeterminate cystic lesion or when considering radiologic differentials. For these reasons, contrast-enhanced CT is generally integrated into the real-world work-up of patients presenting with focal epilepsy or complex headache syndromes. A separate analysis of contrast versus non-contrast imaging would therefore not reflect typical clinical practice and was not feasible within the scope of our study.

Given that perilesional inflammation around calcified lesions is increasingly recognized as a contributor to seizure activity, identifying subtle inflammatory changes is particularly relevant in patients presenting with epilepsy. However, contrast enhancement on CT is a combined result of inflammation, hypervascularization, hyperemia and scarring. A more suitable methodology for the investigation of this discussion point would be MRI studies correlated with clinical data.

5) The definition used in this study (“PWSH were defined as symptoms being progressively worsening in severity…”) is not comparable to the definition used in reference #14, which evaluated severe headaches caused by migraine or tension-type headache, not progressively worsening symptoms. The same applies to the Zambian study. In the discussion, the authors still claim that PWSH is one of the two most common neurological manifestations.

Thank you for your comment, we acknowledge that there are differences in the definitions used for NCC-related headaches and that no single, consistently used definition exists. Therefore whenever referring to earlier work, we have now adapted our wording “progressively worsening severe headaches (PWSH) “ to “headaches”, this in line with our earlier edit of our sentence in the Introduction referring to the findings of reference #1.

6) The authors performed cCT scans in 116 PWSH, 109 with epilepsy, and 15 with both conditions. They exclude the “both” group from analysis but include these individuals in each symptom category. Therefore, each category should include an additional 15 participants, resulting in 131 PWSH and 124 PWE. Why then is the reported number of PWE 126 in the manuscript?

We are truly grateful for this remark and for the reviewer having noticed this error. Indeed, the denominator for the PWE should be 124 throughout. In fact, all the percentages and associated 95%CI were correctly calculated for 124, but unfortunately the denominator was incorrectly presented as 126 instead of 124. This has now been corrected everywhere. We wish to thank the reviewer again for this sharp observation.

**Reviewer #4**

1) Overall, the study shows strong methodological rigor and adequately fulfills the primary requirements for acceptance. The study’s objectives are clearly defined, accompanied by a precise and testable hypothesis. The justification provided underscores the importance and relevance of the research topic. The selected study design is suitable for addressing the proposed objectives. The methodological structure is well-organized, enabling consistent and meaningful data collection. The characteristics of the study population are clearly outlined and appropriate for evaluating the proposed hypothesis. The sample size is adequate to ensure sufficient statistical power, which enhances the reliability and credibility of the results. The statistical approaches employed are suitable and properly executed, consistently reinforcing the study’s conclusions. Data processing is clear, transparent, and methodologically sound. There are no identified issues related to ethical or regulatory adherence. The research complies with established ethical guidelines and good research practices. In conclusion, the study demonstrates solid methodological quality, appropriate analytical procedures, and full ethical compliance, placing it in a strong position for acceptance.

The analysis conducted aligns well with the initial analytical plan, reflecting methodological consistency across the study. The results are presented clearly and thoroughly, allowing readers to readily grasp both the outcomes and their relevance. In addition, the visual materials—such as tables—contribute to the clarity of the data display and reinforcing the interpretation of the findings.

The conclusions are firmly grounded in the data, reflecting the robustness and coherence of the study’s findings. The authors clearly recognize the limitations of their analysis, demonstrating transparency and reinforcing the study’s overall credibility. They also provide a thoughtful discussion on how the results contribute to expanding knowledge in the field, emphasizing the study’s importance within the broader scientific landscape. Additionally, the implications for public health are clearly articulated, highlighting the value of the research in guiding practice and shaping future actions.

Considering that this work has already been reviewed by three reviewers, many of my doubts and questions have been addressed. The modifications made by the authors in relation to the proposed suggestions and comments clarified my doubts and improved the text. For this reason, my opinion is to accept the article for publication.

Thank you for your kind words.
