# Supplementary material for: Prevalence of neurocysticercosis and its characteristics among people with epileptic seizures and progressively worsening severe headaches in 60 villages in three provinces of Burkina Faso
Source: PLoS Negl Trop Dis. 2026 Feb 26;20(2):e0014061. doi: 10.1371/journal.pntd.0014061 (PMC12962496; doi:10.1371/journal.pntd.0014061)
Supplement: Attachment — Submitted filename: Revision_Note_-_Final_auresp_3.docx [file pntd.0014061.s004.docx]

**Revision Note: PNTD-D-25-00377R2**

Dear Editor and Reviewers of PLoS Neglected Tropical Diseases,

We wish to thank you again for your time and thorough review of our revised manuscript. Please find our responses to the new reviewers’ comments and changes suggested below. All changes made within the manuscript were marked using “track changes”. We hope you will now find the manuscript acceptable for publication.

Thank you again,

Best regards,

The authors of the manuscript.

**Reviewers' comments:**

**Reviewer #3**

1) Please clarify in the first sentence on page 18 (“In our study, this prevalence was estimated at a lower 7.6% (10/131, 95%CI: 3.7%-13.6%)” that your study only looked at one type of headaches (PWSH).

We have now edited the text accordingly.

2) In the first sentences of the last paragraph on page 18 (“In our study, all but one PWSH had calcified NCC. This contrasts with a study conducted in Ecuador where the prevalence of calcified NCC in participants with headaches was estimated at 26.3% (15/57) (14). However, a more recent community-based survey showed larger frequency of lifetime headaches prevalence (4.18, 95% CI: 1.79–9.75) among calcified NCC cases compared to controls without headaches (39)”. Please note that your definition of headache was not the same as that used in the two references mentioned.

Thank you for your suggestion, the sentence was adapted accordingly.
